# Electroencephalographic profiles for differentiation of disorders of consciousness

**DOI:** 10.1186/1475-925X-12-109

**Published:** 2013-10-21

**Authors:** Urszula Malinowska, Camille Chatelle, Marie-Aurélie Bruno, Quentin Noirhomme, Steven Laureys, Piotr J Durka

**Affiliations:** 1 Faculty of Physics, University of Warsaw, ul. Hoża 69, Warszawa 00-681, Poland; 2 Coma Science Group, Cyclotron Research Centre, University of Liège, Liège, Belgium; 3 Neurology Dept, University Hospital of Liège, Liège, Belgium

**Keywords:** Electroencephalography, Matching Pursuit, Disorders of consciousness, Minimally conscious state, Vegetative state, Locked-in syndrome

## Abstract

**Background:**

Electroencephalography (EEG) is best suited for long-term monitoring of brain functions in patients with disorders of consciousness (DOC). Mathematical tools are needed to facilitate efficient interpretation of long-duration sleep-wake EEG recordings.

**Methods:**

Starting with matching pursuit (MP) decomposition, we automatically detect and parametrize sleep spindles, slow wave activity, K-complexes and alpha, beta and theta waves present in EEG recordings, and automatically construct profiles of their time evolution, relevant to the assessment of residual brain function in patients with DOC.

**Results:**

Above proposed EEG profiles were computed for 32 patients diagnosed as minimally conscious state (MCS, 20 patients), vegetative state/unresponsive wakefulness syndrome (VS/UWS, 11 patients) and Locked-in Syndrome (LiS, 1 patient). Their interpretation revealed significant correlations between patients’ behavioral diagnosis and: (a) occurrence of sleep EEG patterns including sleep spindles, slow wave activity and light/deep sleep cycles, (b) appearance and variability across time of alpha, beta, and theta rhythms. Discrimination between MCS and VS/UWS based upon prominent features of these profiles classified correctly 87% of cases.

**Conclusions:**

Proposed EEG profiles offer user-independent, repeatable, comprehensive and continuous representation of relevant EEG characteristics, intended as an aid in differentiation between VS/UWS and MCS states and diagnostic prognosis. To enable further development of this methodology into clinically usable tests, we share user-friendly software for MP decomposition of EEG (http://braintech.pl/svarog) and scripts used for creation of the presented profiles (attached to this article).

## Background

### Monitoring brain functions in disorders of consciousness (DOC)

Owing to the progress in medicine, intensive care and technology, more patients survive traumatic accidents and diseases causing brain damage. Some of these patients do not recover from their coma within days and weeks and stay in a state of wakeful unawareness, specified as vegetative state (VS/UWS [1]). Apparent unawareness of some of these patients is merely a consequence of the loss of all motor functions, with full consciousness retained—this state is called locked-in syndrome (LiS [2]). Other patients may reveal at least transient signs of consciousness—this state is defined as minimally conscious state (MCS) and may potentially lead to full recovery [3]. Although all of these states can last for years or even be permanent, it is believed that patients in MCS have much better prognosis for recovery than those in VS/UWS [4]. Precise identification of signs of conscious perception versus sometimes reflex, ambiguous behavior and differentiation between these states is difficult, which causes misdiagnoses of VS/UWS and LiS and of VS/UWS and MCS [5].

Development of recent methods of assessing brain function like positron emission tomography (PET) and functional magnetic resonance imaging (fMRI) disclosed several cases of such misdiagnoses [5,6]. However, even these advanced methods may sometimes provide misleading results, due to significant fluctuations of the state of these patients in time. These changes may be accounted for by long-term monitoring, but PET and fMRI are not well suited for this task. The only technique allowing for long-term monitoring of brain functions in such cases is electroencephalography (EEG, for a review, see [7]). While the “normal” pattern of EEG varies significantly between subjects, assessment of the state of brain functions can be based upon the occurrence of circadian rhythms in EEG. Methodology of their assessment was developed in the field of sleep research.

### Sleep and DOC

Sleep is a state characterized by the absence of response to external stimuli due to transient but reversible period of unconsciousness. Interactions of sleep and consciousness in brain-injured patients are still not known, but, as reported in cases of patients with disorders of consciousness (DOC), presence of some sleep patterns may correlate with diagnosis and prognosis. Early studies on coma suggested that the presence of EEG patterns resembling sleep may be a reliable marker for a favorable outcome [8,9]; it was reported that sleep patterns continue to become more complex during rehabilitation therapy, paralleling patients’ cognitive recovery [10]. EEG pattern which resembles sleep spindle—spindle-coma (SC, activity in 9-14Hz range) was postulated to be an indicator of benign form of coma and, if accompanied by EEG reactivity to noxious stimuli, presage better outcome [9,11]. Some other studies have also indicated that sleep spindles may carry prognostic information. It was shown that the presence of spindle activity after hypoxic or anoxic injury does not always indicate a good outcome, but the absence of spindles or EEG background reactivity does predict a poor outcome [12]. Reference [13] reported better outcome for patients with sleep related patterns in EEG, K-complex responses for stimuli and spontaneous arousals, and the worst in the absence of spontaneous arousals activity.

A more recent study supports these findings in comatose children and concludes that the reappearance of sleep patterns and sleep spindles is a sign of good prognosis. In traumatic coma, these sleep elements are observed more frequently than in anoxic cases [14] and may depend on the time since coma onset. Such activity occurred in 91% of cases if recorded within 1–2 days after injury and in 30% of more prolonged cases (3–12 days) [15]. Another study reports no relationship to the brain region of damage and time since insult [16].

Some studies reported clear relationship between general mental ability and frontal spindle activity, suggesting that spindles may be different between VS/UWS and MCS [17].

Another study [18], where 6 MCS and 5 VS/UWS were analyzed, showed for all patients with MCS an alternating non-rapid eye movement/rapid eye movement sleep pattern and a homoeostatic decline of electroencephalographic slow wave activity through the night in contrast to all patients in a VS/UWS, for which no slow wave sleep or rapid eye movement sleep stages could be identified, and no homoeostatic regulation of sleep-related slow wave activity was observed. Authors suggest that the study of sleep and homoeostatic regulation of slow wave activity may provide a complementary tool for the assessment of brain function in patients with MCS and VS/UWS.

However, a study of 24 h recordings of 10 MCS and 10 VS/UWS [19] suggests a far less clear-cut division: sleep-wake cycles were identified in 50% of MCS and about 30% of VS/UWS; sleep spindles were more predominate in patients who clinically improved in 6 months; slow waves sleep was present in 8/10 - 80% MCS and 3/10 VS/UWS; rapid eye movement was present in all MCS and 3 VS/UWS. In line with these findings, other studies reported the presence of sleep patterns similar to healthy controls in VS/UWS patients [20] and the absence of a correlation between outcome and sleep patterns [21-23].

Altogether, a wide spectrum of sleep disturbances, from almost normal sleep to severe loss and disorganization of sleep, has been reported in DOC (for review see [24]), and the topic is still poorly understood. Also, the spectrum of altered sleep in LiS, as documented in literature, vary from almost normal sleep patterns [25,26], to severe sleep quantity decrease [27-29], disorganized NREM sleep and stage 4 [25,27,28,30], or REM absence [31] depending on the lesions ([32] also reviewed in [24]).

### Automatic analysis of sleep EEG

Analysis of the occurrences of EEG transients like sleep spindles and slow wave activity serves as the basis for construction of the hypnogram—a basic tool in sleep research. In [33] we proposed the first automatic system for creation of hypnograms explicitly based upon the criteria used in the visual analysis of EEG, which still constitutes the golden standard.

**Figure 1:**
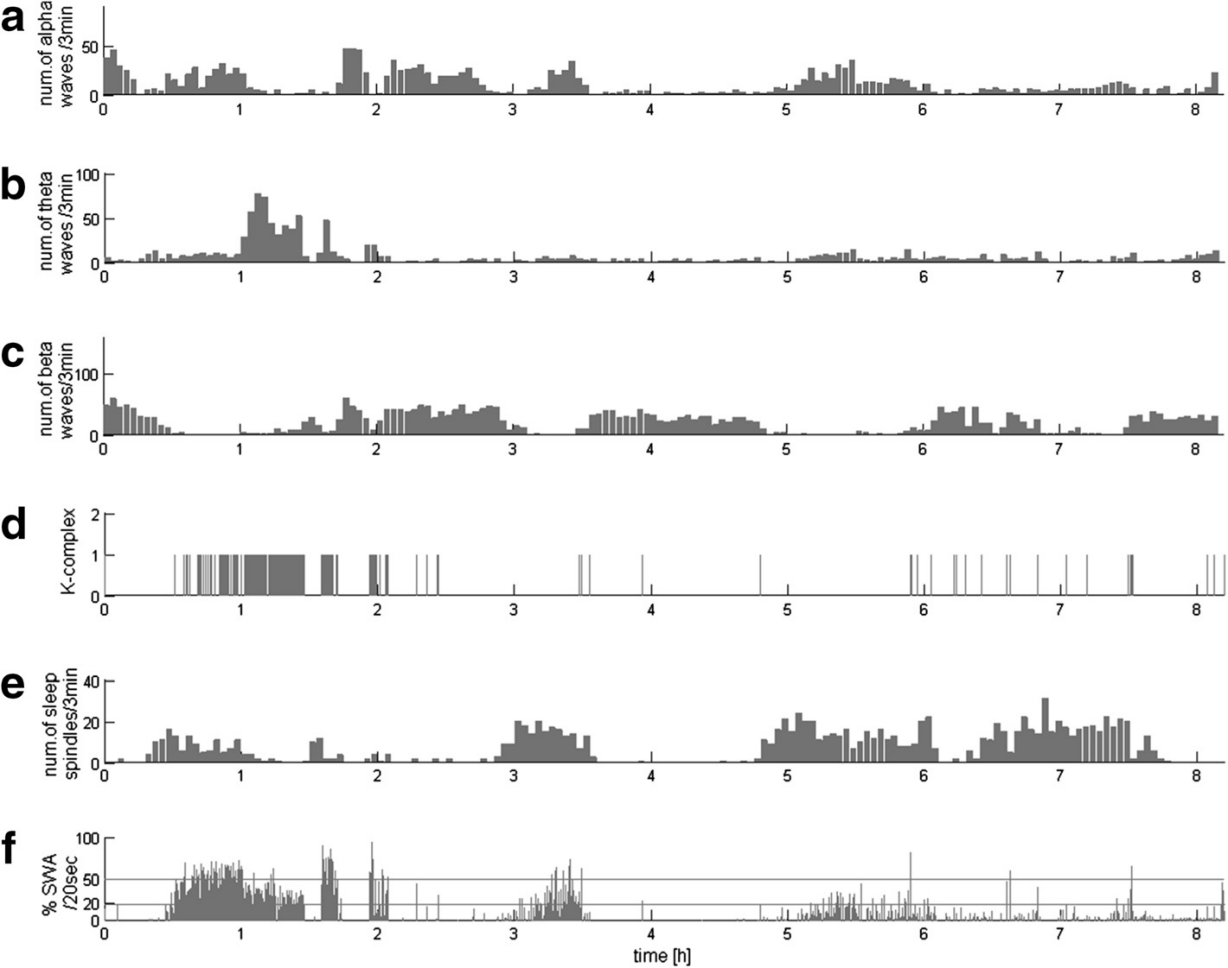
**EEG profile of a sleep recording of control subject**. **(a)**, **(b)**, **(c)** and **(e)** – numbers of waveforms conforming to the criteria defining, correspondingly, alpha, theta and beta waves and sleep spindles, detected per subsequent 3-min epochs. Each vertical line in **(d)** marks one occurrence of a K-complex. Lower panel **(f)** presents percentage of each 20-second epoch occupied by SWA; horizontal lines at 20% and 50% mark the classical criteria for stages 3 and 4 scoring.

This analytical approach, based upon detection of the relevant structures, offers a lot more than the final report of sleep profile in the form of a hypnogram. For example, sleep stages 3 and 4 are defined by the presence of the delta activity in 20-50% and over 50% of the epoch, respectively. To implement this definition explicitly, we assess directly the time span occupied by given structures (as described in section “Selection of EEG structures”). Subsequently, setting the thresholds at 20% and 50% of the length of an epoch (in sleep analysis usually 20 or 30 seconds), we get an explicit detection of stages 3 and 4 based directly upon the classical criteria defined in [34]. Apart from this explicit approach to the classical criteria of scoring sleep stages, these estimates give us a continuous description of the sleep profile as in Figure 1, which has been previously suggested as a welcome enhancement to deal with the shortcomings of the classical sleep staging in 20–30 sec epochs [35-37]. Such EEG profiles can be a valuable tool in the assessment of the circadian pattern of brain activity, which may contain an information important for an assessment of the state of patients in different states of DOC, as presented in this paper. Discussion of the advantages of MP in the analysis of nonstationary EEG can be found in a book [38] and several papers (c.f. [33,39-42]).

Above mentioned occurrence of well described micro-structures (K-complexes, spindles), transients and waveforms which form stages [34], and cycles accompanied repeatedly more or less by arousals, is typical for a normal sleep pattern. On the contrary, in DOC the existence of normal sleep stages and polysomnographic elements is a matter of debate. Some authors suggest persistence of sleep stages in some more “evolved state”, whereas others refute this. Problems in definition of wakefulness and sleep in DOC are due to the uncertainty whether the oscillations recorded by EEG reflect still the same cellular mechanisms as in normal physiological sleep. For example, large amplitude slow waves in coma may not necessarily indicate “slow-wave” sleep (SWS) and deep non-rapid eye movement (NREM) sleep, as they do in normal sleeping individuals, because generalized slowing in delta and theta ranges (continuous delta activity) is a phenomenon generally observed in coma. The same kind of observed spindles-coma (SC) may not necessary represent the same mechanism as sleep spindles in normal sleep process. Therefore, classical criteria for sleep staging [34] cannot be directly applied to recordings from patients with DOC.

In this paper we propose automatic constructions of EEG profiles, in a way maximally compatible with classical sleep scoring criteria, allowing for monitoring comatose patients’ brain electrical activity and focusing on changes in micro- and macrostructure of sleep and awake patterns.

## Methods

### Experimental data

This paper presents analysis of EEG recordings of 32 brain-injured patients with disorders of consciousness. Among these 32, 20 patients were diagnosed as a MCS. Their average age was 33 (range 5–56), mean of time from insult 560 days (range 25–2633), 11 of these 20 MCS cases were in a chronic state (> 1 year post insult); 9 females and 12 males. Next 11 patients were diagnosed in a VS/UWS, their average age was 55 (range 3–75), mean time from insult 347 days (range 8–2348); 3 of these cases were in a state (> 1 year post insult); 6 females and 5 males. Three of the 11 patients with VS/UWS and 13 of the 20 with MCS had traumatic etiology, respectively. Dataset include also one recording of a patient (female) in LiS who had a rombencephalitis, examined about 2 years after insult. All patients were evaluated at the University Hospital of Liège, in Liège, Belgium. Two patients from the original pool were excluded because of short time of acquisition. Other nine patients had to be excluded because of technical problems (computer problems not complete data, missed clinical information). Clinical assessment and diagnosis were based on the standardized Coma Recovery Scale-Revised [43]. Detailed description of patient’s characteristics is reported in columns 1–5 of Table 1. Dataset contained also 5 recordings from healthy subjects for reference (3 females and 2 males, age 24–26). This study was approved by the Ethics Committee of the Faculty of Medicine of the University of Liège.

**Table 1:** Patients information and summary of EEG profiles

**1**	**2**	**3**	**4**	**5**	**6**	**7**	**8**	**9**	**10**	**11**	**12**
**Patient #**	**Age**	**Etiology**	**Days since onset**	**Behavioral diagnosis**	**SS**	**δ (SWA)**	**Spikes**	**Other activi. αβθ**	**Cycles light- deep sleep**	**Variability**	**Other comments (’small’- small, not significant number of detections)**
1	36	CVA	2348	VS/UWS	-	-	+	-	-	-	Mostly isoelectrical signal
2	29	TBI	2633	MCS	+	-	+	αβθ	-	+	Small δ
3	15	Rhombencephalitis	712	LIS	+	+	+	αβθ	+	+	All activity
4	34	TBI	1015	MCS	+	+	+	αβ	-	+	Small δ, small θ, big β
5	56	Anoxia	392	MCS	-	-	+	β	-	-	Poor activity, few β, small Variab
6	25	TBI + hypoxia	310	MCS	-	-	+	β	-	-	Small δ, small Variab
7	38	TBI	516	MCS	+	+	-	αβθ	+	+	Nice β -sleep relationship, small ampl SS
8	36	anoxia	547	MCS	-	-	-	β	-	-	Poor small θ andδ, small Variab
9	30	TBI	585	MCS	-	+	+	αβθ	-	+	Small SS, small cycles
10	30	TBI	564	VS/UWS	-	+	-	-	-	-	Poor activity; small other, small ampl δ, small spikes
11	5	TBI	1113	MCS	+	+	+	αβθ	+	+	Like opposite homeost. cycle
12	31	TBI	141	MCS	+	+	-	αβθ	+	+	Lots of α, small spikes, small ampl δ
13	25	TBI	1285	MCS	+	-	+	αβθ	+	-	Poor activity, small Variab
14	28	ADE	713	MCS	-	+	-	αβθ	+	+	Few α, small spikes
15	61	Toxoplasmose	16	VS/UWS	+	-	-	αβθ	-	-	Small δ, smalls Cycles, small Variab
16	31	Hematoma	44	MCS	+	+	+	αβθ	+	+	Small α, small ampl SS, small spikes
17	61	Anoxia	119	VS/UWS	-	+	+	β	-	-	Few θ, small SS, small Cycles, small Variab
18	48	TBI	238	MCS	+	+	+	αβθ	+	+	No clear wake-sleep periods, small ampl δ
19	17	TBI	25	MCS	+	+	+	αβθ	+	+	No wake-sleep cycles
20	53	TBI	62	MCS	+	+	+	αβθ	+	+	All activity
21	31	TBI	224	MCS	+	+	-	αβθ	+	+	Small spikes
22	61	Brainstem hemmorrh.	35	VS/UWS	-	-	-	βθ	-	-	Poor variability
23	74	TBI	15	VS/UWS	-	-	+	αθ	-	-	NoVariab across 24 h, no β, a lot of spikes, small ampl SS, small ampl δ
24	45	SH	361	MCS	+	+	-	αβθ	+	+	Continuous small θ, small α, small spikes
25	55	Cardiac arrest	25	MCS	+	+	-	αβθ	+	+	Contin α, small θ, small spikes
26	19	TBI	214	MCS	+	+	+	αβθ	+	+	Lot of θ
27	21	TBI	756	MCS	+	+	-	αβθ	+	+	Lot of β, small θ
28	35	Anoxia (infection)	522	VS/UWS	-	+	+	βθ	+	-	Lot of β, small θ, small SS, small C, small Variab
29	45	Hypoglycemia	108	VS/UWS	-	-	+	αβθ	-	+	Lot of β, θ, small Cycles, small SS, small δ, isoelectrical
30	75	TBI	8	VS/UWS	+	-	+	αθ	-	-	Contin. α, contin.θ, small δ, small Cycles, small other
31	62	Pontine Hemorrhage	49	VS/UWS	-	-	-	αθ	-	-	Contin. α, continθ, small spikes
32	70	Meningoencephalitis	31	VS/UWS	-	-	-	αβ	-	-	Mostly contin. α, small SS, small δ, small Variab

Columns 1–5: patients clinical information; columns 6–12: summary of EEG profiles computed for patients with disorders of consciousness; TBI: traumatic brain injury; CVA: cardiovascular arrest; SH: subarachnoïdale hemorrhage due to aneurysm disruption; ADE: Acute demyelinating encephalomyelitis. δ: delta; θ: theta activity; α: alpha activity; β: beta activity; SS: sleep spindles, +: present; -: absent.

EEG was recorded using one of the following systems:

1) high density EEG (Electrical Geodesics), 256 electrodes sampled at 500Hz, referenced to Cz with simultaneous, synchronized video-taped recordings to confirm the patients behavior With this system 15 MCS, 7 VS/UWS, 1 LiS, and 5 control subjects were examined.

2) polysomnography recording performed with a V-Amp amplifier (Brain Products), 12 EEG channels localized according to the 10–20 system plus EMG, two EOG, and ECG, with sampling rate also 500Hz. With this system 5 MCS and 4 VS/UWS were examined.

Minimal time of acquisition was 9 hours. All recordings include night time. For each patient, EEG electrode C3 or C4 (referenced to mastoid A2 or A1), as recommended for sleep scoring [34,44] and bandpass filtered in 0.5-40 Hz was used for further analysis.

### Matching Pursuit

Matching Pursuit (MP) is a suboptimal solution to the problem of optimal representation of a function in a redundant dictionary, proposed by Mallat and Zhang [45]. When used with dictionary of Gabor functions to decompose time series, it offers an adaptive time-frequency parameterization of the structures present in the analyzed signal. The procedure can be summarized as follows:

1. We start by creating a huge, redundant dictionary of candidate waveforms for representation of structures possibly occurring in the signal. For the time-frequency analysis of signals we use dictionaries composed of sines with Gaussian envelopes, called Gabor functions, which reasonably represent waxing and waning of oscillations.

2. From this dictionary we choose only those functions, which fit the local signal structures. This choice is based on a maximum inner product of the function and signal’s residuum, left after subtracting the functions fitted in previous iterations (Eq. (1)). In such a way, the width of the analysis window is adjusted to the local properties of the signal.

3. Local adaptivity of the procedure is somehow similar to the process of visual analysis, where an expert tends to separate local structure and assess their characteristics. Owing to this local adaptivity, MP is the only signal processing method returning explicit time span of detected structures.

Using equations, the above reads:

(1) $$\left\{\begin{array}{c} R^{0}x=x\\ R^{n}x=〈R^{n}x,g_{\gamma_{n}}〉g_{\gamma_{n}}+R^{n+1}x\\ g_{\gamma_{n}}=\arg\max_{g_{\gamma_{n}}\in D}\left|〈R^{n}x,g_{\gamma_{i}}〉\right| \end{array}\right)$$

where *x* is the decomposed signal and *R*^*n*^ is the *n*th residue left after subtracting results of previous iterations. As a result we get an expansion

(2) $$x\approx \sum_{n=0}^{M-1}〈R^{n}x,g_{\gamma_{n}}〉g_{\gamma_{n}}$$

Apart from the high resolution and adaptivity to the local signal structures, MP offers a unique advantage of explicit parametrization in terms of not only frequency and amplitude, but also time span for each detected structure. This feature was employed in [39] for explicit parametrization of slow waves and, together with other structures, for construction of an automatic sleep stager based explicitly on the classical scoring criteria [33].

In this study MP decomposition was performed on subsequent 20s epochs of C3 or C4 derivation of all the available recordings. Details of the MP algorithm and the freely available software package used for this decomposition are given in [46]. From this decomposition, relevant structures were identified using criteria explained in the following section.

### Selection of EEG structures

From the set of functions chosen by the MP algorithm for representation of the EEG time series we can automatically select those corresponding to particular structures of interest, by setting the ranges for their amplitude, frequency and width in time (sometimes also phase). Criteria used in this work for selection delta waves, sleep spindles, K-complexes, epileptiform spikes, alpha, theta and beta waves are given in Table 2.

**Table 2:** Criteria for selection of EEG structures

	**Frequency** [**Hz**]	**Duration** [**s**]	**Min. amplitude** [**μV**]
Delta waves	0.2 – 4	>0.5	70
Sleep spindles	11 – 15	0.5-2.5	12
K-complexes*	0.05 – 2.5	0.3 - 1.5	100
Theta waves	4 – 8	> 1	15
Alpha waves	8 – 12	> 1.5	5
Beta waves	15 – 25	> 0.5	4
Spikes*	0.2 – 7	0.05 - 0.35	50

Parameters of functions from MP decomposition, used for automatic detection of relevant waveforms. *For K-complexes an additional constraint on the phase, enforcing a negative deflection and condition for the amplitude to exceed also the background amplitude by factor 1.5, for spikes or the amplitude to exceed also the average background amplitude by factor 2.

Selection and quantification of relevant structures, which explicitly takes into account also their time widths and amplitudes, is significantly more selective and sensitive than the classically employed spectral estimators, as was presented e.g. in [41]. It is a natural consequence of performing the discrimination in three weakly correlated dimensions (amplitude, time width and frequency) rather than just one (frequency) and allows also for a better separation of relevant structures from those due to the artifacts. Previously published MP-based studies of normal sleep [33,40,42] were performed without any artifact-rejection, which significantly increases the repeatability and objectivity of the proposed procedure, since different schemes of artifact detection may bias the choice of artifact-free epochs and hence also the final results. Also in this study no artifact-contaminated epochs were excluded from analysis, and relevant structures were detected from MP decompositions of the whole available recordings.

### EEG profiles

Application of the above discussed criteria to the results of MP decomposition (Eq. (2)) provides automatically a detailed description of all the relevant EEG structures, present in the analyzed recording. This procedure not only saves dozens of hours of tedious work of experienced electroencephalographers, but also provides strict repeatability. From such a database of relevant structures we can produce various types of reports and graphs—in this paper we concentrate on their time course.

Figure 1 presents example of continuous description of EEG activity from normal subject across all-night recording. Alpha, theta, and beta activities, as well as sleep spindles (subplots a, b, c, e), are represented by the number of occurrences in subsequent 3 min epochs. Each K-complex is represented in subplot d by vertical line in the time of its occurrence. Finally, for slow waves we compute the percent of time which these waves occupy in every subsequent 20-second period (subplot f), a parameter used in analysis of deep sleep stages by rules for normal sleep scoring [34].

These profiles constructed for sleep EEG of a control subject reveal gradual changes in the activity of the slow waves. Sleep spindles occur in a reverse relationship to the occurrence of delta waves. Occurrences of K-complexes correspond to those of sleep spindles and especially in the first sleep cycle also to SWA. Beta waves are dominating at the beginning of the sleep (or in the period leading to the onset of sleep) and at the end of night. In this recording beta activity increases also in some periods of the night, but these detections may be associated with muscle artifacts. According to a well-known observation, beta waves show a clear trend reversed in the presence of delta waves at the beginning of sleep, a trend that fades with the fall in SWA activity during the night. Theta waves coincide with the detected delta waves, sometimes slightly ahead of the slow waves. Alpha waves are observed across almost whole time of the recordings, with a high concentration at the beginning of sleep and around the Stage 2, of which can be associated with the appearance of sleep spindles—activity in the band close to the frequency of alpha waves.

All the analyzed recordings of control subjects were characterized by the occurrence of all the waveforms and appearance of sleep cycles. Rates of particular activities revealed differences across night, and gradual changes were observed in proportions of deep/light sleep–from predominance of deep sleep in the first part of night to REM sleep during second half of the night.

### Reproducible research and availability of software

Software used for this article (except for the Matlab® commercial package) is freely available. Downloading, installation and use of this software, as well as mathematical and numerical optimizations used in the mp5 implementation of MP, are covered in details in [46]. MP decomposition of EEG with a user friendly GUI is embedded in the Svarog (Signal Viewer, Analyzer and Recorder on GPL) package, which can be freely downloaded from http://braintech.pl/svarog. From the MP decomposition, EEG profiles presented in this work were computed by a set of Matlab® scripts, attached in Additional file 1. This archive contains also decomposition of the signal used for Figure 1, and so allows to recreate this figure.

## Results and discussion

### EEG profiles of DOC patients

**Figure 2:**
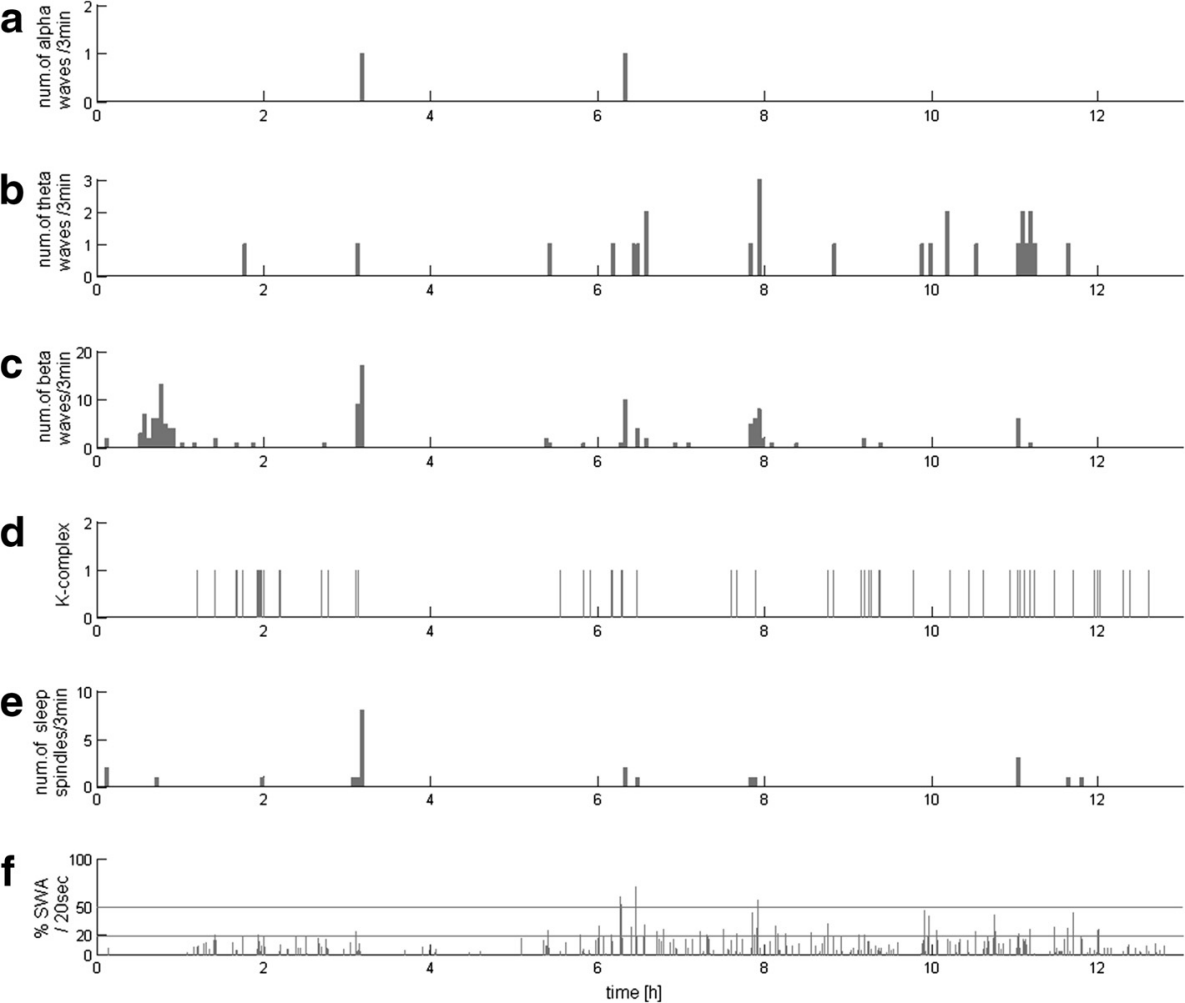
**EEG profile for all**-**night VS**/**UWS recording with residual detection of all analyzed activity.** Subplots organized as in Figure 1. **(a)**, **(b)**, **(c)** and **(e)** – numbers of alpha, theta and beta waves and sleep spindles, detected per subsequent 3-min epochs, **(d)** markers of occurrence of K-complexes, **(f)** percentage of each 20-second epoch occupied by SWA; horizontal lines at 20% and 50% mark the classical criteria for stages 3 and 4 scoring.

**Figure 3:**
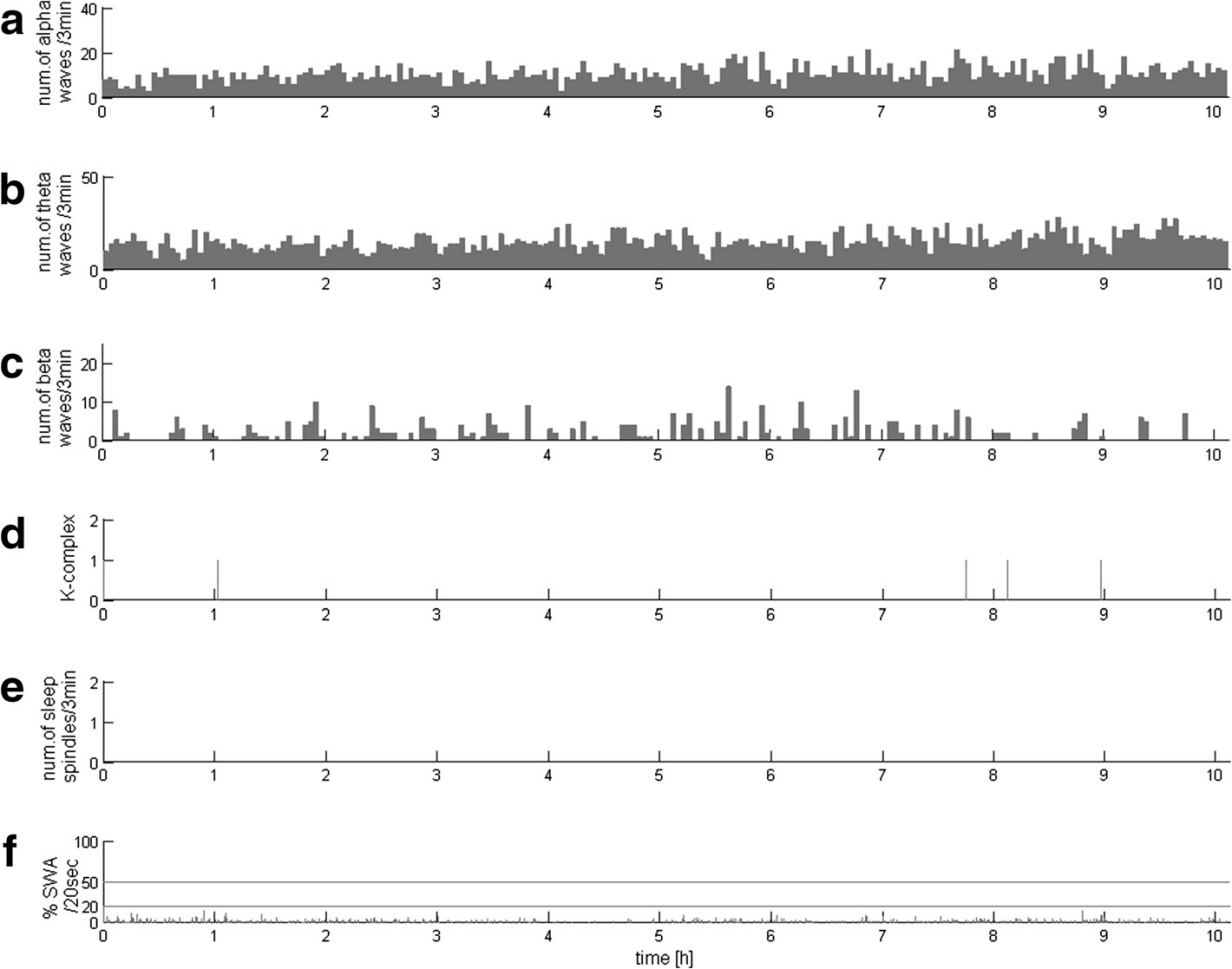
**EEG profile for all**-**night VS**/**UWS recording with the lack of sleep spindles and poor SWA**, **but the presence of alpha**, **theta**, **beta waves**- **in a large proportion but continuous and not differentiated activity**. Subplots organized as in Figure 1. **(a)**, **(b)**, **(c)** and **(e)** – numbers of alpha, theta and beta waves and sleep spindles, detected per subsequent 3-min epochs, **(d)** markers of occurrence of K-complexes, **(f)** percentage of each 20-second epoch occupied by SWA; horizontal lines at 20% and 50% mark the classical criteria for stages 3 and 4 scoring.

**Figure 4:**
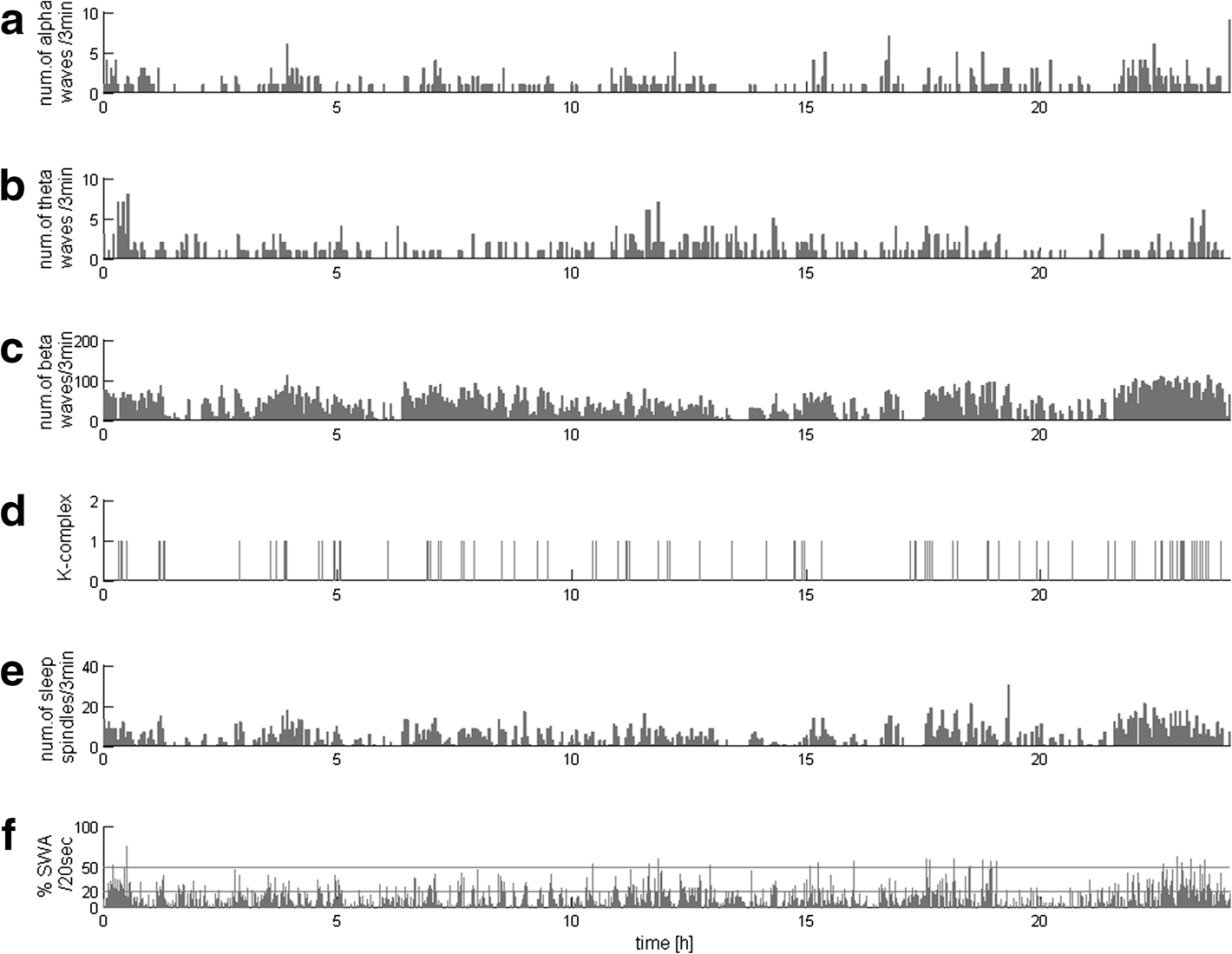
**EEG profile of 24 hours recording**. An example of continuous monitoring of patient’s EEG activity during long period of time. Recording with many sleep spindles detected but no typically concentrated during night, not correlated with the slow waves and without variation across 24 hours. Subplots organized as in Figure 1. **(a)**, **(b)**, **(c)** and **(e)** – numbers of alpha, theta and beta waves and sleep spindles, detected per subsequent 3-min epochs, **(d)** markers of occurrence of K-complexes, **(f)** percentage of each 20-second epoch occupied by SWA; horizontal lines at 20% and 50% mark the classical criteria for stages 3 and 4 scoring.

**Figure 5:**
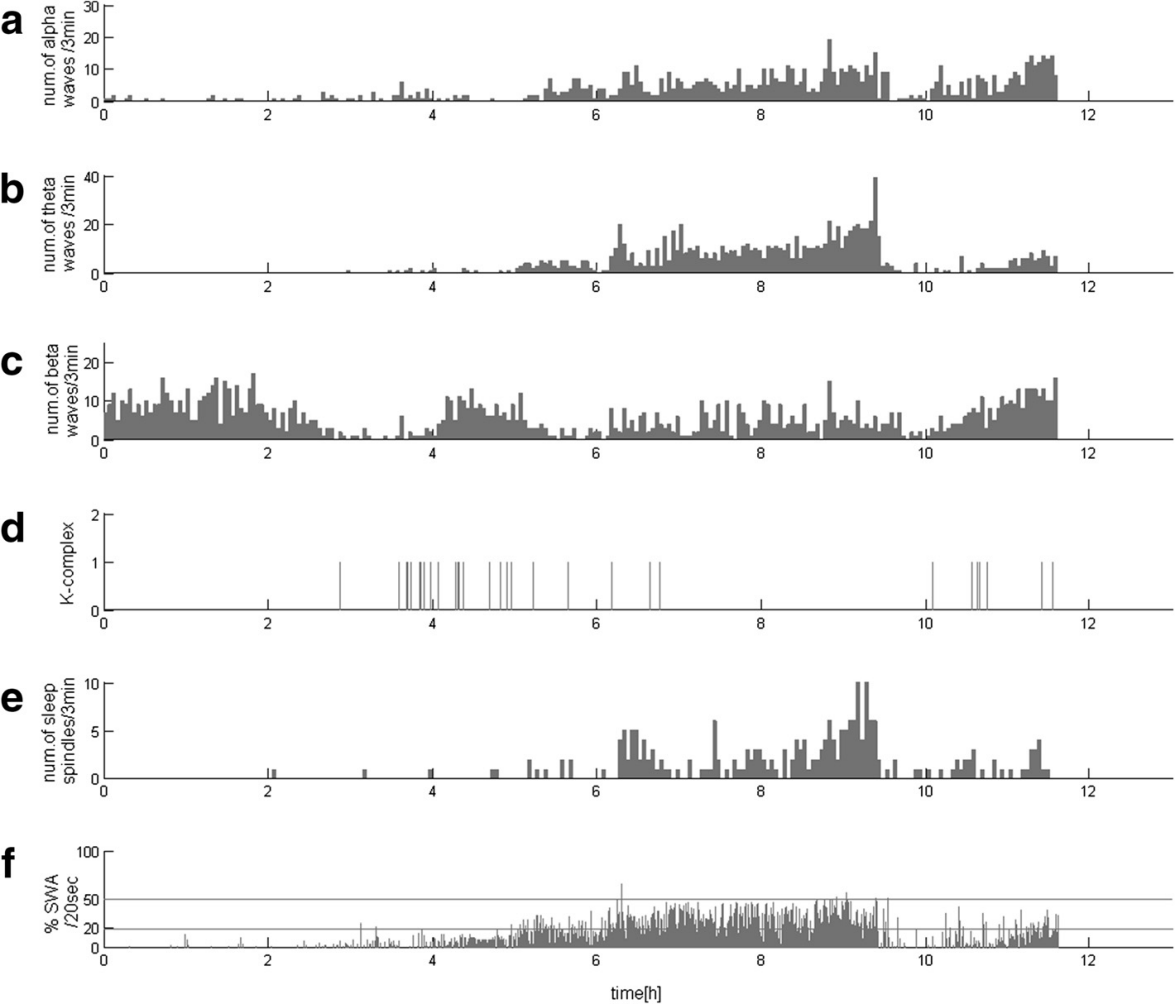
**EEG profile of all**-**night MCS recording.** In this case we observe increase of the number of sleep spindles in specific intervals during the night, and their inverse relationship to slow waves and other activity, corresponding to the pattern of normal sleep profile. Subplots organized as in Figure 1. **(a)**, **(b)**, **(c)** and **(e)** – numbers of alpha, theta and beta waves and sleep spindles, detected per subsequent 3-min epochs, **(d)** markers of occurrence of K-complexes, **(f)** percentage of each 20-second epoch occupied by SWA; horizontal lines at 20% and 50% mark the classical criteria for stages 3 and 4 scoring.

In the same way as the above example of normal sleep from the control group, EEG recordings of all the 32 DOC patients were analyzed by means of the method proposed in the previous section. These profiles revealed great variety between patients—which may have been expected, but hereby it was presented using an objective and parametric method. Representative examples of these 32 profiles are presented in Figures 2, 3, 4 and 5.

None of these profiles has shown the typical profile of normal sleep homeostatic decline. The reason for this may be that their daily cycle is not associated with the cycle of day and night.

As mentioned in the Background section, the most important in diagnosis and prognosis of DOC patients are occurrences in EEG of the sleep patterns of the occurrence of structures such as sleep spindles, slow waves, and their variability across time. Figures 2, 3, 4 and 5 present the variability of these patterns, computed for different patients. Characteristics of these profile vary from residual detection of all analyzed activity (Figure 2), through the lack of sleep spindles and poor SWA (Figure 3), but presence of alpha, theta, and beta waves—in a large proportion but continuous and not differentiated activity, characterized by abundance of sleep spindles (Figure 4), but no variation across 24 hours and correlation with the slow waves, to the case (Figure 5) where the spindles are increased in the specific intervals during the night, and their reverse relationship to slow waves and other activity corresponds to the pattern of a normal sleep profile.

Figure 4 presents 24 hours EEG profile as an example of continuous monitoring of patients’ EEG activity during longer periods of time.

### Assessment of predominant features of EEG profiles and statistics

Automatically constructed plots like Figures 2, 3, 4 and 5 provide a novel insight into the time evolution of classical EEG features, traditionally assessed via visual analysis or spectral methods with significantly lower time resolution. Apart from the elements used for sleep staging (slow waves, sleep spindles and spikes that is interictal epileptiform activity), we evaluated also other parameters, including:

1. appearance of alternating cycles of deep and light sleep (present(+)/absent(−))

2. degree of variability of brain activities across time (‘variability’:

present(+)/absent(−))

3. presence of alpha, theta and beta waves (“other activities”): (αβθ)

These features were assessed visually for each patient, based upon plots like those presented in Figures 1, 3, 4 and 5. For each patient and each parameter, binary assessment of whether given feature is present or not was marked in columns 6–11 of Table 1. The last parameter (presence of alpha, theta and beta) was classified for further statistical analysis as present if all of them were detected, absent if none were identified in patient’s EEG and partial if some of “other activity”, for example only beta, or only alpha or theta were present.

Above mentioned EEG features (columns 6-11 of Table 1) were correlated with the clinical data (columns 2–5 of Table 1) available for each of the patients:

•behavioral diagnosis (according to the Coma Recovery Scale-Revised, (VS/UWS)/MCS/LIS, [43]).

•interval since insult: less than 1 year or chronic (> 1 year post insult).

•etiology: traumatic or other non-traumatic etiology.

For statistical evaluation of these data, Pearson’s chi-squared test was used. It was performed separately for pairs consisting of one of the clinical parameters describing patients’ state (columns 3–5) and one of the EEG-derived parameters (columns 6–11). The results were thresholded for significance at p < 0.05 and corrected for multiple comparison (marked **). Results which proved significant only in separate comparisons are marked with “*”.

**Figure 6:**
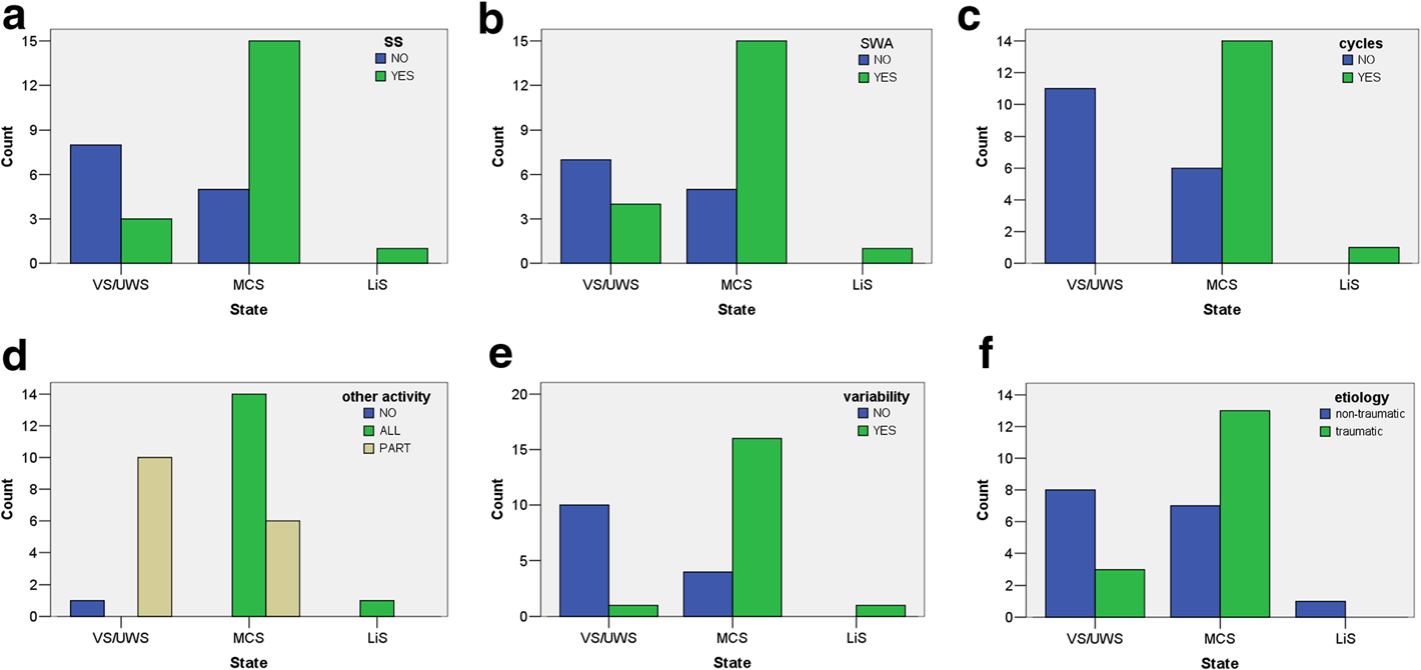
**Results of correlation of patient states (behavioral diagnosis) and analyzed EEG parameters.** Bars plots of statistically significant correlation of patients state and: SS (p = 0.01*) fig. **a)**, SWA (p = 0.035*) fig. **b)**, occurrence of sleep cycles (p < 0.001**) fig. **c)**, other EEG activity (p = 0.001**) fig. **d)** variability of these all activity across time (p < 0.001**) fig. **e)** and etiology (p = 0.044*) fig. **f)**.

Figure 6 presents statistically significant relations between patients state and EEG-derived parameters:

1. sleep spindles are more likely found in MCS patients (p = 0.01*): they occurred in 27% of VS/UWS, but in 75% MCS and in LiS patient.

2. delta waves were found in 36% of patients with VS/UWS and in 75% of patients with MCS and for the LiS patient, (p = 0.035*).

3. cycles of light and deep sleep were not detected in VS/UWS, detected in 70% MCS and for the LiS patient, (p < 0.001**).

4. “other activities” (alpha, beta, theta) was absent in all patients with VS/UWS but present in 70% if patients with MCS and for the LiS patient. Single frequency band (e.g., only beta, only alpha or theta) was present in 90% of patients with VS/UWS and in 30% of patients with MCS, (p = 0.001**).

5. variability of detected activity across time were detected for 9% VS/UWS, 80% MCS and for the LiS patient, (p < 0.001**).

**Figure 7:**
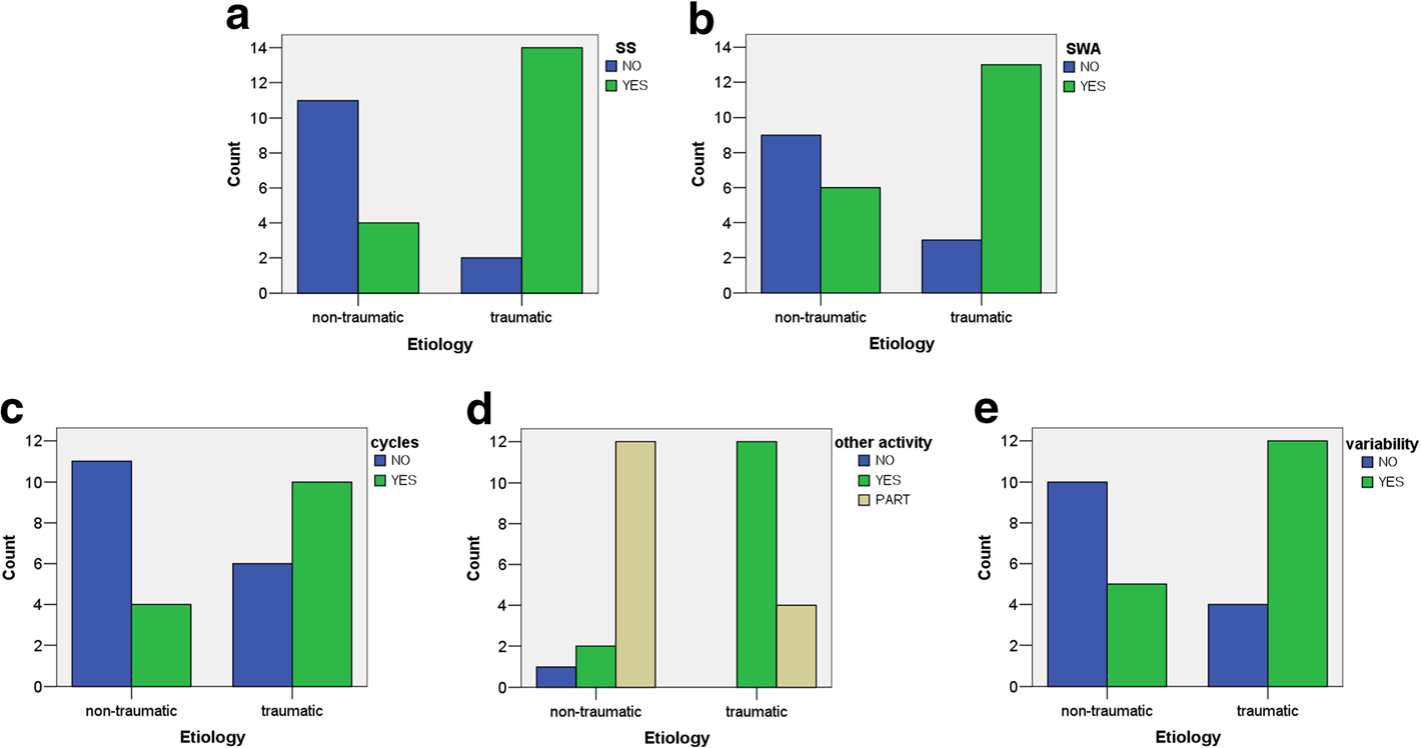
**Results of correlation of analyzed EEG parameters and etiology and time from insult for each patient.** Statistically significant correlation are presented on bars plots: occurrence of SS and etiology (p = 0.001**) fig. **a)**, occurrence of SWA and etiology (p = 0.018*) fig. **b)**, occurrence of sleep cycles (p = 0.045*) fig. **c)**, other EEG activity (p = 0.002**) fig. **d)** and variability of these all activity across time versus etiology (p = 0.02*) fig. **e)**.

Figure 7 presents statistically significant relations between etiology and EEG-derived parameters:

1. sleep spindles were observed in 78% of patients with traumatic etiology and only in 22% of non-traumatic etiology, (p = 0.001**).

2. delta waves are also more common for traumatic brain injury patients - observed in 68% post-traumatic, and only in 32% non-traumatic etiology, (p = 0.018*).

3. sleep cycles in 71% post-traumatic, and only in 29% non-traumatic etiology, (p = 0.045*).

4. all „other activities“(alpha, beta and theta) were present in 86% of cases for traumatic and 14% of non-traumatic patients’ etiology. Only some of the „other activities “were detected for 25% post-traumatic and in 75% of non-traumatic, (p = 0.002**).

5. variability of detected activity across time were found in 71% of patients with traumatic brain injury and only in 29% in non-traumatic etiology patients, (p = 0.021*).

Less patients diagnosed VS/UWS were found in cases after traumatic brain injury than non-traumatic. Within the analyzed group of patients there is a significant dependence between patients diagnosed state and etiology of DOC (p = 0.044*). 27% patients in VS/UWS have traumatic etiology, 73% other. In MCS 65% of patients etiology were traumatic, 35% other. This is an important factor which will have to be taken into account in following studies, as etiology has an influence on patients‘ outcome and can be a confounding factor here.

Finally, discriminant analysis carried out on the basis of behavior diagnosis of patients states indicated that the most important features for MCS and VS/UWS discrimination are: occurrence of sleep cycles, variability of detected activity across time and occurrence of SS and SWA. Occurrence of interictal spikes is not significant factor. Discrimination between MCS and VS/UWS based upon prominent features of these profiles classified correctly 87% of cases, 3 diagnosed MCS were classified as VS/UWS and 1 VS/UWS as MCS. When adding etiology information as an extra parameter in the analysis of correlation between patients state and EEG-derived parameters, the same effect was observed suggesting that etiology is not a confounding factor (on the border of significance). Only separate comparison between patient’s states and etiology of patient’s disorders indicated p = 0.044* suggesting significant correlation.

As compared to the study from Giubilei and colleagues [21] who reported the presence of sleep patterns similar to healthy controls in 9 out of 10 traumatic acute VS/UWS, we observed poorer sleep patterns in a majority of VS/UWS as compared to MCS patients. Moreover, other studies showed the absence of a correlation between outcome and sleep pattern in VS/UWS patients [21,22]. We observed a correlation between the level of consciousness and etiology and sleep cycles, traumatic and MCS patients being more likely to show complex sleep patterns as compared to non-traumatic and VS/UWS patients. It has been suggested in the literature that etiology and level of consciousness influence outcome in DOC patients [4,47]; our results support the potential of automatized EEG sleep recordings as a complementary diagnostic and/or prognostic tool for assessing DOC patients at bedside.

## Conclusions

This study investigates the clinical interest of an automatic sleep analyzer for assessing sleep preservation in a group of patients with DOC. Using this system, we report significant differences in the occurrene of sleep waves characteristics between conscious patients (MCS and LIS) and unconscious patients (VS/UWS). We also found an etiology effect, traumatic patients being more likely to show preserved EEG sleep-like activities, which agrees with previous literature [47,48]. Altogether, these results highlight the applicability of an automatic sleep analyzer to study clinical population such as patients with DOC and to improve our knowledge about the diagnosis and prognosis in this population. Moreover, this would also have a major impact for clinical settings, were sleep examinations currently remain time-consuming and subjective.

## Abbreviations

DOC: Disorders of consciousness; EEG: Electroencephalography; fMRI: Functional magnetic resonance imaging; LiS: Locked-in syndrome; MCS: Minimally conscious state; VS/UWS: Vegetative state; MP: Matching pursuit; PET: Positron emission tomography.

## Competing interests

The authors declare that they have no competing interests.

## Authors’ contributions

UM conceived and designed the study, computed, interpreted, and assessed statistical significance of the presented results, and together with PJD wrote this article. PJD proposed the application of MP-based EEG profiles to continuous assessment of sleep and then application of this methodology to assessment of DOC. CC, MB, QN and SL provided the experimental data, clinical information and contributed to the discussion of the clinical aspects. All authors read and approved the final manuscript.

## Supplementary Material

Additional file 1 **Matlab® scripts recreating Figure** 1**.** This package contains a set of Matlab® scripts *.m and one binary file norma_sleep_C3-LM_128_smp.b, containing MP decomposition of the sample recording analyzed in Figure 1. This decomposition was computed using the 5th version (mp5) of the matching pursuit software, developed at the University of Warsaw. It can be downloaded from http://braintech.pl/svarog. To reproduce the figure, run “plot_figure1.m“. Parameters defining EEG structures are in files corresponding to their names (alpha_mp5.m, SS_mp5.m etc.), and can be modified directly in the code – see file “HOWTO.txt” enclosed in the archive. Click here for file
